# Complete mitochondrial genome sequence of *Thryssa kammalensis* (Clupeiformes, Engraulidae)

**DOI:** 10.1080/23802359.2016.1167637

**Published:** 2016-04-19

**Authors:** Liangjie Zhao, Qigen Liu

**Affiliations:** The Key Laboratory of Aquatic Genetic Resources and Utilization (AGRU) of the Ministry of Agriculture, Shanghai Ocean University, Shanghai, P. R. China

**Keywords:** Engraulidae, molecular systematics, mitochondrial genome, Thryssa kammalensis

## Abstract

In this paper, the complete mitochondrial DNA (mtDNA) sequence of *Thryssa kammalensis* was determined. The mitochondrial genome is 16,884 bp in length, including 13 protein-coding genes, 2 ribosomal RNAs (rRNAs), 22 transfer RNAs (tRNAs), and a non-coding control region as those found in other vertebrates, with the gene identical to that of typical vertebrates. The overall base composition of the heavy strand is 31.07% A, 24.82% T, 28.33% C, and 15.78% G, with an AT bias of 55.89%. Phylogeny of *T. kammalensis* suggested more close relationship with *Lycothrissa crocodilus* and *Setipinna taty*. The complete mitogenome of this species can provide a basic data for the studies on population history, molecular systematics, phylogeography, stock evaluation, and conservation genetics.

*Thryssa kammalensis* (Madura anchovy) is a commercially important Clupeiformes fish species in China, which belongs to the family Engraulidae and is distributed in the western part of the Indian Ocean and the Pacific Ocean. Mitochondrial DNA is used as a genetic marker for species identification, genetic structures, systematic, and phylogeography in various organisms due its maternal inheritance, high mutation rate, and rapid evolution (Avise et al. 1987). In this study, the specimens of *T. kammalensis* were collected from Shanwei, Guangdong Provience, China. The specimen was stored in the Key Laboratory of Aquatic Genetic Resources and Utilization (AGRU) of the Ministry of Agriculture in Shanghai Ocean University, and named ‘TK01’ as sample code. The genomic DNA was extracted by modified PCI method followed by ethanol precipitation (Sambrook & Russel 2001). Primers were designed on the basis of mitogenome sequences of *Setipinna taty* (GenBank Accession No. KC439458), a closely related species of *T. kammalensis* (Lavoue et al. 2010). The complete mitochondrial DNA sequence of *T. kammalensis* was firstly determined (GenBank Accession No. KU761588) using PCR and sequencing. According to our data, the complete mitochondrial genome of *T. kammalensis* was 16,884 bp in length, including 13 protein-coding genes, 2 ribosomal RNA (rRNA) genes, 22 transfer RNA (tRNA) genes, 1 L-strand replication origin (OL), and one control region (D-loop). The overall nucleotide composition was 31.07% A, 24.82% T, 28.33% C, and 15.78% G, with an AT bias of 55.89% which is similar to other fishes (Cheng et al. 2010, 2011a; Jin et al. 2012). The order, direction, locations of the genes and gene coding strand in the mitogenome were nearly identical to those in other Engraulidae species (Zhang & Sun 2013; Li et al. 2014; Zhang & Xian 2014) and the usual vertebrate consensus (Xu et al. 2011; Cheng et al. 2011b; Shi et al. 2012). ND6 gene and eight tRNA genes were encoded on the light(L) strain and the remaining genes were encoded on the heavy (H) strain. For protein-coding genes, 12 were initiated with the orthodox ATG except for COI with GTG, and the three types of termination codons revealed were TAA (ND1, ND4L, ND5, COI, ATP8, ATP6, COIII, ND3 and ND6), T(COII, Cytb, and ND4), TAG(ND2). The incomplete stop codons were presumably completed by post-transcriptional polyadenylation (Ojala et al. 1981). All the tRNA genes of *T. kammalensis* fold into a typical cloverleaf structure, except tRNA^ser^ (AGY) which lacks a dihydrouridine arm identified by tRNA scan SE 1.21 (Lowe & Eddy 1997). The two ribosomal RNA genes, 12S rRNA (949bp) and 16S rRNA (1681bp), are located between tRNA^Phe^ and tRNA^Leu^ (UUR) and are separated by tRNA^Val^ gene. The control region is located between tRNA^pro^ and tRNA^phe^ and is 1223 bp in length.

Phylogenetic analysis was done using MEGA 6.0 (Tamura et al. 2013) program with 13 complete mitogenomes under family Engraulidae and one sequence of *Clupea harengus* as the out-group. We found that the phylogeny of *T. kammalensis* suggested more close relationship with *Lycothrissa crocodilus* and *S. taty*, consistent with the traditional evolutionary position (Figure 1).

**Figure 1.**
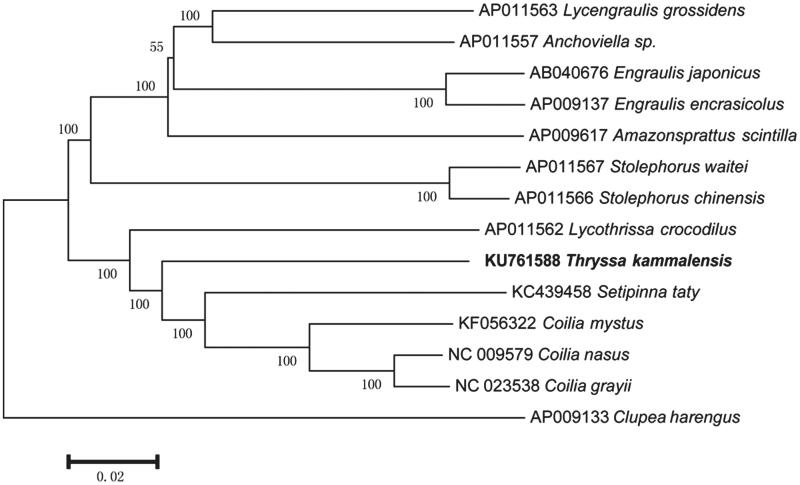
Neighbour-joining (NJ) tree showing relationships among *Thryssa kammalensis* and related families.
